# Response to: ‘when man got his mtDNA deletions?’

**DOI:** 10.1111/acel.12230

**Published:** 2014-06-04

**Authors:** Sean D Taylor, Jesse J Salk, Jason H Bielas

**Affiliations:** 1 Translational Research Program, Public Health Sciences Division, Fred Hutchinson Cancer Research Center1100 Fairview Ave, Seattle, WA, 98109, USA; 2 Department of Medicine, University of Washington Medical Center1959 NE Pacific St, Seattle, WA, 98195, USA; 3 Department of Pathology, University of Washington Medical Center1959 NE Pacific St, Seattle, WA, 98195, USA; 4 Human Biology Division, Fred Hutchinson Cancer Research Center1100 Fairview Ave, Seattle, WA, 98109, USA

We appreciate the ardor and detail with which Popadin *et al*. have examined our data. The primary concern raised in their accompanying commentary regards our supposition that the age-associated increase in mtDNA deletions in human brain is disproportionately driven by clonal expansion of existing mutant genomes rather than *de novo* events. Our conclusion was based on the observation that, while the absolute frequency of deletions unambiguously increases with age, the abundance of unique deletions identified by deep sequencing does not.

The authors of the critique astutely note that the number of mitochondrial genomes used for the emulsion PCRs in this study was systematically lower in older individuals than younger individuals and argue that this variable input confounds proper determination of sample mutational diversity. They then take a direct multiplicative approach to normalize the number of unique deletions we identified to an extrapolated population of 10^10^ input genomes and arrive at a contradictory conclusion whereby the frequency of unique deletions does increase with age.

The concern about unequal inputs is justified and does reasonably challenge one of the biological conclusions of our study. The variation in mtDNA input was intentional, as the higher deletion frequency in older individuals necessitated relatively greater dilutions to achieve a single molecule concentration in the proper Poisson range for droplet PCR. We reasoned that because a similar amount of DNA was extracted and homogeneously mixed from each tissue sample, that larger or smaller samplings from a uniform population would retain the representative mutational diversity of the original sample. In retrospect, we realize this cannot be unconditionally assumed; yet for the same reason, the adjusted calculations presented by Popadin and colleagues are similarly incorrect by the following logic.

Consider two hypothetical extreme scenarios where 10^10^ mtDNA genomes are isolated from a sample: in one case, only a handful of independent deletion events have occurred, but thousands of copies of each deletion are present in the sample by virtue of clonal expansion of the mutant genome within a cell and through cell division; in a second scenario, every single mutant genome derives from a unique, independent deletion event with no subsequent expansion. If one-tenth of each population is then inputted into an emulsion PCR, in both cases, the absolute frequency of mutant molecules, calculated as the number of positive droplets divided by the PCR input (10^9^ mtDNA genomes), will accurately reflect the *absolute* deletion frequency of the original larger population. However, after disrupting and deep sequencing the emulsion to quantify *unique* deletion frequency, a very different situation arises with the two scenarios. In the first, where many copies of a few distinct deletion events are present, the chance of the 10% subsample capturing at least a few copies of every independent deletion event in the larger population is essentially assured and the calculated unique deletion frequency will accurately represent that of the larger population. In the second case, because all molecules in the larger population are unique, sequencing of a 10% subsampling will underestimate the true abundance of unique deletions by 90%, and a tenfold correction factor is necessary to yield the true value.

Our original calculation made the former assumption, whereas the correctional approach of the commenters makes the latter. In reality, neither extreme view is likely to be correct. The several orders of magnitude difference we see in number of sequencing reads belonging to each unique deletion family supports the notion that there is substantial variation in relative expansion of different deletions. As such, we concede the probability of relative undercounting in older-aged samples and the likelihood that, in addition to clonal expansion of existing deletions, *de novo* deletions contribute to the observed increase with age, although not to the degree that the commenters assert. Given that the relative distribution of deletion clone size is unknown and appears to vary between samples, it is impossible to impartially normalize the existing data through any calculation.

The correct way to carry out future such experiments will be to hold the total number of inputted genomes fixed for all samples and adjust the total volume of the emulsion PCR as necessary to maintain a proper Poisson distribution of deleted molecules. An additional nuance with this approach is that when disrupting emulsions containing a different total number of positive droplets (i.e., samples with differing absolute mutation frequencies) for sequencing, it will be critical to dedicate a proportional amount of sequencer lane capacity to each sample based on droplet count to ensure that each positive droplet across samples has an equal probability of being represented.

We appreciate this productive dialogue and an outside perspective revealing an important aspect of normalization that was not readily apparent to us when developing the Digital Deletion Detection (3D) technology. We continue to recognize and stress that many additional variables such as spatial and anatomical variation within individual brains and distribution of deletions among different cell types (i.e., neurons vs. supporting glial cells) remain unknown and merit further detailed investigation. Our primary intention with this work is to present a powerful new tool to the scientific community, and we anticipate that much of the potential for discovery lies ahead. We look forward to future studies, by others and ourselves, using 3D to advance an improved collective understanding of the temporal dynamics of mitochondrial genetic changes throughout life and their role in aging and disease.

